# Sex Differences in Subclinical Atherosclerosis and Systemic Immune Activation/Inflammation Among People With Human Immunodeficiency Virus in the United States

**DOI:** 10.1093/cid/ciac767

**Published:** 2022-09-14

**Authors:** Markella V Zanni, Borek Foldyna, Sara McCallum, Tricia H Burdo, Sara E Looby, Kathleen V Fitch, Evelynne S Fulda, Patrick Autissier, Gerald S Bloomfield, Carlos D Malvestutto, Carl J Fichtenbaum, Edgar T Overton, Judith A Aberg, Kristine M Erlandson, Thomas B Campbell, Grant B Ellsworth, Anandi N Sheth, Babafemi Taiwo, Judith S Currier, Udo Hoffmann, Michael T Lu, Pamela S Douglas, Heather J Ribaudo, Steven K Grinspoon

**Affiliations:** Metabolism Unit, Massachusetts General Hospital and Harvard Medical School, Boston, Massachusetts, USA; Cardiovascular Imaging Research Center, Department of Radiology, Massachusetts General Hospital and Harvard Medical School, Boston, Massachusetts, USA; Metabolism Unit, Massachusetts General Hospital and Harvard Medical School, Boston, Massachusetts, USA; Department of Microbiology, Immunology, and Inflammation and Center for NeuroVirology and Gene Editing, Temple University Lewis Katz School of Medicine, Philadelphia, Pennsylvania, USA; Metabolism Unit, Massachusetts General Hospital and Harvard Medical School, Boston, Massachusetts, USA; Yvonne L. Munn Center for Nursing Research, Massachusetts General Hospital and Harvard Medical School, Boston, Massachusetts, USA; Metabolism Unit, Massachusetts General Hospital and Harvard Medical School, Boston, Massachusetts, USA; Metabolism Unit, Massachusetts General Hospital and Harvard Medical School, Boston, Massachusetts, USA; Department of Biology , Boston College, Chestnut Hill, Massachusetts, USA; Department of Medicine, Duke Global Health Institute and Duke Clinical Research Institute, Duke University, Durham, North Carolina, USA; Division of Infectious Diseases, Ohio State University Medical Center, Columbus, Ohio, USA; Division of Infectious Diseases, University of Cincinnati College of Medicine, Cincinnati, Ohio, USA; Division of Infectious Diseases, University of Alabama at Birmingham School of Medicine, Birmingham, Alabama, USA; Division of Infectious Diseases, Icahn School of Medicine at Mount Sinai, New York, New York, USA; Department of Medicine, Division of Infectious Disease, University of Colorado—Anschutz Medical Campus, Aurora, Colorado, USA; Department of Medicine, Division of Infectious Disease, University of Colorado—Anschutz Medical Campus, Aurora, Colorado, USA; Department of Medicine, Division of Infectious Diseases, Weill Cornell Medicine, New York, New York, USA; Division of Infectious Diseases, Department of Medicine, Emory University School of Medicine, Atlanta, Georgia, USA; Division of Infectious Diseases and Center for Global Health, Northwestern University, Chicago, Illinois, USA; Division of Infectious Diseases, David Geffen School of Medicine, University of California Los Angeles, Los Angeles, California, USA; Cardiovascular Imaging Research Center, Department of Radiology, Massachusetts General Hospital and Harvard Medical School, Boston, Massachusetts, USA; Cardiovascular Imaging Research Center, Department of Radiology, Massachusetts General Hospital and Harvard Medical School, Boston, Massachusetts, USA; Duke University Research Institute, Duke University School of Medicine, Durham, North Carolina, USA; Center for Biostatistics in AIDS Research, Harvard T. H. Chan School of Public Health, Boston, Massachusetts, USA; Metabolism Unit, Massachusetts General Hospital and Harvard Medical School, Boston, Massachusetts, USA

**Keywords:** HIV, coronary atherosclerosis, inflammation, women, reproductive aging

## Abstract

**Background:**

Among people with HIV (PWH), sex differences in presentations of atherosclerotic cardiovascular disease (ASCVD) may be influenced by differences in coronary plaque parameters, immune/inflammatory biomarkers, or relationships therein.

**Methods:**

REPRIEVE, a primary ASCVD prevention trial, enrolled antiretroviral therapy (ART)–treated PWH. At entry, a subset of US participants underwent coronary computed tomography angiography (CTA) and immune phenotyping (n = 755 CTA; n = 725 CTA + immune). We characterized sex differences in coronary plaque and immune/inflammatory biomarkers and compared immune-plaque relationships by sex. Unless noted otherwise, analyses adjust for ASCVD risk score.

**Results:**

The primary analysis cohort included 631 males and 124 females. ASCVD risk was higher among males (median: 4.9% vs 2.1%), while obesity rates were higher among females (48% vs 21%). Prevalence of any plaque and of plaque with either ≥1 visible noncalcified portion or vulnerable features (NC/V-P) was lower among females overall and controlling for relevant risk factors (RR [95% CI] for any plaque: .67 [.50, .92]; RR for NC/V-P: .71 [.51, 1.00] [adjusted for ASCVD risk score and body mass index]). Females showed higher levels of IL-6, hs-CRP, and D-dimer and lower levels of Lp-PLA2 (*P* < .001 for all). Higher levels of Lp-PLA2, MCP-1, and oxLDL were associated with higher plaque (*P* < .02) and NC/V-P prevalence, with no differences by sex. Among females but not males, D-dimer was associated with higher prevalence of NC/V-P (interaction *P* = .055).

**Conclusions:**

Among US PWH, females had a lower prevalence of plaque and NC/V-P, as well as differences in key immune/inflammatory biomarkers. Immune-plaque relationships differed by sex for D-dimer but not other tested parameters.

**Clinical Trial Registration.** ClinicalTrials.gov; identifier: NCT0234429 (date of initial registration: 22 January 2015).

## Teaser

Among US persons with HIV (PWH), females demonstrated a lower prevalence of subclinical coronary artery disease (CAD), as well as differences in key immune/inflammatory biomarkers. Understanding sex-specific immune drivers of subclinical CAD will facilitate appropriately tailoring cardiovascular disease–preventive therapies to PWH.

Epidemiologic studies have highlighted both sex differences in human immunodeficiency virus (HIV)–attributable risks of myocardial infarction (MI) [1] and sex differences in MI presentations among people with HIV (PWH) in the United States [2]. In the Partners Healthcare Database cohort, the adjusted relative risk (RR) of MI was 2.98 among females with versus without HIV compared with 1.4 among males with versus without HIV [1]. Meanwhile, in the Center for AIDS Research Network of Integrated Clinical Systems (CNICS) cohort, females with HIV who experienced MI more frequently presented with type 2 MI (54% type 2 vs 46% type 1), whereas males with HIV who experienced MI more frequently presented with type 1 MI (59% type 1 vs 41% type 2) [2].

Understanding mechanisms underlying sex differences in HIV-attributable MI risks and sex differences in MI presentations among PWH is crucial to optimizing sex- and HIV-specific atherosclerotic cardiovascular disease (ASCVD) prevention strategies. Presently, such mechanisms are incompletely understood. A small study of PWH residing in a single US region has suggested a lower burden of subclinical atherosclerotic plaque and high-risk morphology plaque among females as compared with males [3]. In concert, studies of PWH across US regions have demonstrated sex differences in patterns of persistent systemic immune activation/inflammation [4]. Thus far, no large study has contemporaneously assessed sex differences in subclinical coronary atherosclerotic plaque phenotypes and in systemic immune activation/inflammation biomarkers among a diverse primary prevention cohort of antiretroviral therapy (ART)–treated PWH living in the United States.

In the present study, we leverage baseline data from the Randomized Trial to Prevent Vascular Events in HIV (REPRIEVE), an international randomized controlled trial testing whether pitavastatin calcium versus placebo prevents major adverse cardiovascular events (MACE) among ART-treated PWH [5]. As part of an embedded substudy, a subset of US REPRIEVE participants underwent coronary computed tomography (CT) angiography (CTA) and immune phenotyping at baseline and 2 years after randomization [6]. Our first exploration of REPRIEVE baseline data in 755 substudy participants revealed that 48.7% had any coronary plaque, 40% had plaque with 1 or more visible noncalcified portion, and 22.8% had plaque with vulnerable features [7]. Levels of monocyte chemoattractant protein-1 (MCP-1), interleukin-6 (IL-6), lipoprotein-associated phospholipase A2 (Lp-PLA2), and oxidized LDL (oxLDL) were associated with coronary plaque, and relationships held for IL-6 and Lp-PLA2 after adjustment for traditional risk indices and HIV-specific parameters [7].

The work presented here builds on our previous findings in pursuit of the following objectives: (1) to characterize sex differences in subclinical coronary atherosclerotic plaque among PWH, (2) to characterize sex differences in soluble immune/inflammatory biomarkers among PWH, and (3) to evaluate among PWH whether relationships between immune/inflammatory biomarkers and coronary plaque differ by sex. To establish whether female sex influences subclinical coronary atherosclerosis in ways not fully captured by the traditional assignment of sex-based CVD risk, analyses adjusted for ASCVD risk score.

## METHODS

REPRIEVE enrolled 7770 PWH, age 40–75 years on ART and with low-to-moderate traditional cardiovascular disease (CVD) risk [8]. Inclusion and exclusion criteria are as previously described [5]. Each clinical research site obtained institutional review board/ethics committee approval and other applicable regulatory entity approvals. Informed consent was obtained from all participants. Participants enrolled into REPRIEVE at any of 31 US sites were offered an opportunity to co-enroll in the substudy (barring contraindication to coronary CTA) [6]. A total of 805 co-enrolled between May 2015 and February 2018 [7].

### Clinical Data

Data on demographic parameters, traditional cardiometabolic risk parameters, and HIV-specific parameters were collected, as previously described [5–8]. Natal sex and race were self-reported. Among cis-gender female participants, postmenopausal status was determined through application of an algorithm described in Supplementary Figure 1. ASCVD risk score was calculated using the Pooled Cohorts Equation [9].

### Coronary Computed Tomography Angiography

#### Image Acquisition

Site selection procedures and quality-control measures for CTA data acquisition and radiation were as previously described [6, 7]. All coronary CTA scans were acquired on at least 64-slice CT scanners using standardized protocols according to the Society of Cardiovascular CT (SCCT) guidelines [10]. A central REPRIEVE CT core laboratory reviewed the scans for completeness, quality, and radiation dose [6, 7].

#### Presence, Extent, and Composition of Coronary Artery Disease

Contrast-enhanced coronary CTA scans were assessed for the presence, extent, and composition of atherosclerotic plaques using the standard 18-segment SCCT model [11]. Plaques were characterized based on the presence or absence of at least 1 visible noncalcified portion (noncalcified plaque [NCP]). Plaques were also characterized based on the presence or absence of at least 1 vulnerable plaque feature (VP): low attenuation (plaque with portions that have attenuation <30 Hounsfield units), positive remodeling (remodeling index >1.1), or napkin ring sign (plaque with low central attenuation and ring-like peripheral high attenuation) [12]. Coronary artery calcium (CAC) score was quantified on non-contrast cardiac CT using a modified Agatston method [13]. All CT datasets were analyzed by 1 of 3 trained central REPRIEVE core laboratory readers on a dedicated workstation, with good interreader agreement [7]. Participants’ scans were initially evaluated in real time by the CT core laboratory for critical stenosis, with information on critical stenosis relayed back to sites [7].

### Immune/Inflammatory Biomarker Data

Participants’ fasting, cryopreserved plasma samples were shipped to Temple University (Philadelphia, PA) for centralized quantification of levels of immune/inflammatory biomarkers using enzyme-linked immunosorbent assay kits: soluble CD14 (sCD14; R&D), soluble CD163 (sCD163; IQ Products), MCP-1 (R&D), high-sensitivity IL-6 (R&D), Lp-PLA2 (R&D), and oxLDL (Mercodia) [7]. D-dimer was measured using the HemosIL D-dimer HS 500 kit on the ACL TOP (Werfen). Fasting, cryopreserved serum samples were shipped to Quest Diagnostics for quantification of high-sensitivity C-reactive protein (hs-CRP) by commercially available assays.

### Statistical Methods

Comparisons between groups were performed using a Wilcoxon rank-sum test for continuous variables and chi-square test for categorical variables. The RR of plaque outcomes for females (compared with males) was assessed using log binomial regression with and without adjustment for the following: (1) ASCVD risk score; (2) ASCVD risk score and body mass index (BMI); (3) ASCVD risk score and metabolic syndrome; (4) individual CVD risk factors, including age, race, cigarette smoking, and HDL cholesterol; (5) individual CVD risk factors and BMI; or (6) ASCVD risk score and HIV-specific risk factors (CD4 count, abacavir exposure, protease inhibitor exposure). Different effects of ASCVD risk level by sex were assessed via interaction effects. Immune biomarker outcomes (on the log_2_ scale) were compared between females and males with estimates back-transformed to a geometric mean ratio for an estimate of the average relative difference, using linear regression adjusting for ASCVD risk score or ASCVD risk score and BMI. Adjusted log binomial regression assessed the association of biomarkers with plaque outcomes. Biomarkers were log_2_ transformed and standardized to estimate the RR of plaque for each 25% increase in the level of the marker. Interaction terms for sex were assessed for each biomarker–plaque relationship in the overall model, and sex-stratified analyses for biomarker–plaque relationships were performed. Inference was guided with a 2-sided 5% false-positive error rate without adjustment for multiple comparisons; sex-stratified analyses considered the consistency of effect sizes and 95% confidence intervals (CIs). Statistical analyses were performed using SAS version 9.4M7 (SAS Institute).

## RESULTS

### Study Population

Of the 805 participants enrolled in the REPRIEVE substudy, 755 had a baseline coronary CTA suitable for assessment for the presence and composition of coronary atherosclerosis [7]. Among these participants, 725 also provided blood samples yielding immune/inflammatory biomarker data.

### Baseline Demographic and Clinical Parameters: Overall and Sex-Stratified

Baseline demographic and clinical parameters for the 755 participants (124 females, 631 males) with CTA data are shown in Table 1. Median age was comparable among females and males. Females (compared with males) had markedly lower ASCVD risk scores (median: 2.1% vs 4.9%, respectively). Obesity (BMI ≥30 kg/m^2^) was more prevalent among females (48%) than among males (21%). Modest between-group differences were also noted with respect to race and CD4 count. Many other demographic parameters, cardiometabolic parameters, comorbid conditions, and HIV-related parameters were comparable.

**Table 1.** Demographic and Clinical Characteristics

	Participants With Plaque Outcomes		
Characteristics	Total (N = 755)	Female (n = 124)	Male (n = 631)
Demographic and behavioral			
Age, median (Q1, Q3), y	51 (47, 55)	50 (46, 55)	51 (47, 55)
ȃ40–49 y, n (%)	322 (43%)	56 (45%)	266 (42%)
ȃ50–59 y, n (%)	376 (50%)	57 (46%)	319 (51%)
ȃ60+ y, n (%)	57 (8%)	11 (9%)	46 (7%)
Gender identity, n (%)			
Cisgender	722 (96%)	121 (98%)	601 (95%)
Transgender spectrum	15 (2%)	0 (0%)	15 (2%)
Not reported	18 (2%)	3 (2%)	15 (2%)
Race,^a^ n (%)			
White	406 (54%)	41 (33%)	365 (58%)
Black or African American	267 (35%)	70 (56%)	197 (31%)
Asian	10 (1%)	2 (2%)	8 (1%)
Other	72 (10%)	11 (9%)	61 (10%)
Ethnicity,^b^ n (%)			
Hispanic or Latino	182 (24%)	33 (27%)	149 (24%)
Not Hispanic or Latino	563 (75%)	91 (73%)	472 (75%)
Unknown	10 (1%)	0 (0%)	10 (2%)
Smoking status, n (%)			
Current	181 (24%)	31 (25%)	150 (24%)
Former	235 (31%)	37 (30%)	198 (31%)
Never	337 (45%)	55 (45%)	282 (45%)
Substance use, n (%)			
Current	16 (2%)	3 (2%)	13 (2%)
Former	367 (49%)	57 (46%)	310 (49%)
Never	369 (49%)	63 (51%)	306 (49%)
Cardiovascular and metabolic			
ASCVD risk score, median (Q1, Q3), %	4.5 (2.6, 6.8)	2.1 (0.9, 3.7)	4.9 (3.1, 7.3)
0–<2.5, n (%)	175 (23%)	72 (58%)	103 (16%)
2.5–<5, n (%)	247 (33%)	33 (27%)	214 (34%)
5–10, n (%)	286 (38%)	19 (15%)	267 (42%)
>10, n (%)	47 (6%)	0 (0%)	47 (7%)
BMI, median (Q1, Q3), kg/m²	26.9 (24.3, 30.1)	29.6 (25.3, 32.7)	26.6 (24.0, 29.4)
<18.5 kg/m², n (%)	8 (1%)	1 (1%)	7 (1%)
18.5–24.9 kg/m², n (%)	247 (33%)	28 (23%)	219 (35%)
25–29.9 kg/m², n (%)	307 (41%)	35 (28%)	272 (43%)
30+ kg/m², n (%)	193 (26%)	60 (48%)	133 (21%)
Prior statin use, n (%)	59 (8%)	6 (5%)	53 (8%)
Hypertension, n (%)	244 (32%)	42 (34%)	202 (32%)
Diabetes, n (%)	3 (<0.5%)	0 (0%)	3 (<0.5%)
Cholesterol,^c^ median (Q1, Q3), mg/dL	183 (160, 209)	192 (164, 221)	181 (159, 205)
Triglycerides,^c^ mg/dL			
Triglycerides,^c^ median (Q1, Q3), mg/dL	110 (81, 162)	102 (82, 138)	112 (80, 168)
≤400 mg/dL, n (%)	737 (98%)	124 (100%)	613 (98%)
401–<500 mg/dL, n (%)	7 (1%)	0 (0%)	7 (1%)
500+ mg/dL, n (%)	6 (1%)	0 (0%)	6 (1%)
LDL cholesterol,^c^ median (Q1, Q3), mg/dL	105 (88, 127)	108 (87, 127)	105 (88, 127)
<70 mg/dL, n (%)	72 (10%)	14 (11%)	58 (9%)
70–130 mg/dL, n (%)	503 (68%)	82 (66%)	421 (68%)
130–160 mg/dL, n (%)	131 (18%)	23 (19%)	108 (17%)
160+ mg/dL, n (%)	38 (5%)	5 (4%)	33 (5%)
HDL cholesterol,^c^ median (Q1, Q3), mg/dL	47 (38, 59)	59 (47, 71)	45 (37, 56)
Glucose,^c^ median (Q1, Q3), mg/dL	92 (85, 98)	89 (83, 96)	92 (86, 98)
Comorbid conditions, n (%)			
Chronic active HBV	18 (2%)	0 (0%)	18 (3%)
Chronic active HCV	24 (3%)	3 (2%)	21 (3%)
HIV-related health history			
Years since HIV diagnosis, median (Q1, Q3)	15 (9, 22)	16 (9, 23)	15 (9, 21)
Nadir CD4 count, n (%)			
<50 cells/mm³	163 (22%)	24 (19%)	139 (22%)
50–199 cells/mm³	218 (29%)	42 (34%)	176 (28%)
200–349 cells/mm³	202 (27%)	31 (25%)	171 (27%)
350+ cells/mm³	148 (20%)	23 (19%)	125 (20%)
Unknown	24 (3%)	4 (3%)	20 (3%)
Years of ART use, n (%)			
<5	120 (16%)	22 (18%)	98 (16%)
5–10	199 (26%)	27 (22%)	172 (27%)
10+	436 (58%)	75 (60%)	361 (57%)
HIV-related health at REPRIEVE entry			
CD4 count, n (%)			
<350 cells/mm³	112 (15%)	12 (10%)	100 (16%)
350–499 cells/mm³	148 (20%)	31 (25%)	117 (19%)
500+ cells/mm³	495 (66%)	81 (65%)	414 (66%)
HIV-1 RNA, n (%)			
<LLQ	658 (88%)	113 (94%)	545 (87%)
LLQ–< 400	71 (10%)	5 (4%)	66 (11%)
400+	16 (2%)	2 (2%)	14 (2%)
ART regimen, n (%)			
NRTI + INSTI	334 (44%)	57 (46%)	277 (44%)
NRTI + NNRTI	197 (26%)	32 (26%)	165 (26%)
NRTI + PI	127 (17%)	18 (15%)	109 (17%)
NRTI-sparing	22 (3%)	4 (3%)	18 (3%)
Other	75 (10%)	13 (10%)	62 (10%)
Abacavir exposure, n (%)	253 (34%)	47 (38%)	206 (33%)
Abacavir exposure, median (Q1, Q3), years	3.0 (1.2, 6.7)	4.0 (1.0, 6.4)	3.0 (1.2, 6.8)
Protease inhibitor exposure, n (%)	464 (62%)	78 (63%)	386 (61%)
Protease inhibitor exposure, median (Q1, Q3), years	6.7 (3.4, 11.0)	7.3 (4.0, 10.7)	6.6 (3.3, 11.0)

All statistics are calculated out of participants with data collected. Missing data: Smoking status (n = 2); Substance use (n = 3); Cholesterol (n = 5); Triglycerides (n = 5); LDL Cholesterol (n = 11); HDL Cholesterol (n = 5); Glucose (n = 8); HIV-1 RNA (n = 10). Abbreviations: ART, antiretroviral therapy; ASCVD, atherosclerotic cardiovascular disease; BMI, body mass index; HBV, hepatitis B virus; HCV, hepatitis C virus; HIV, human immunodeficiency virus; INSTI, integrase strand transfer inhibitor; LLQ, lower limit of quantification; NNRTI, non-nucleoside reverse-transcriptase inhibitor; NRTI, nucleoside reverse-transcriptase inhibitor; PI, protease inhibitor; Q, quartile; REPRIEVE, Randomized Trial to Prevent Vascular Events in HIV.

“Other” race includes participants self-identifying as native or indigenous to the enrollment region, >1 race (with no single race noted as predominant), or of unknown race.

Ethnicity presented per National Institutes of Health definition.

Fasting labs at entry. LDL cholesterol is derived as follows: calculated LDL reported by the laboratory if triglycerides ≤ 400 mg/dL. Direct LDL reported by the laboratory if triglycerides >400 mg/dL and <500 mg/dL. Missing if triglycerides ≥ 500 mg/dL.

### Plaque Outcomes: Sex-Stratified

**Figure 1.**
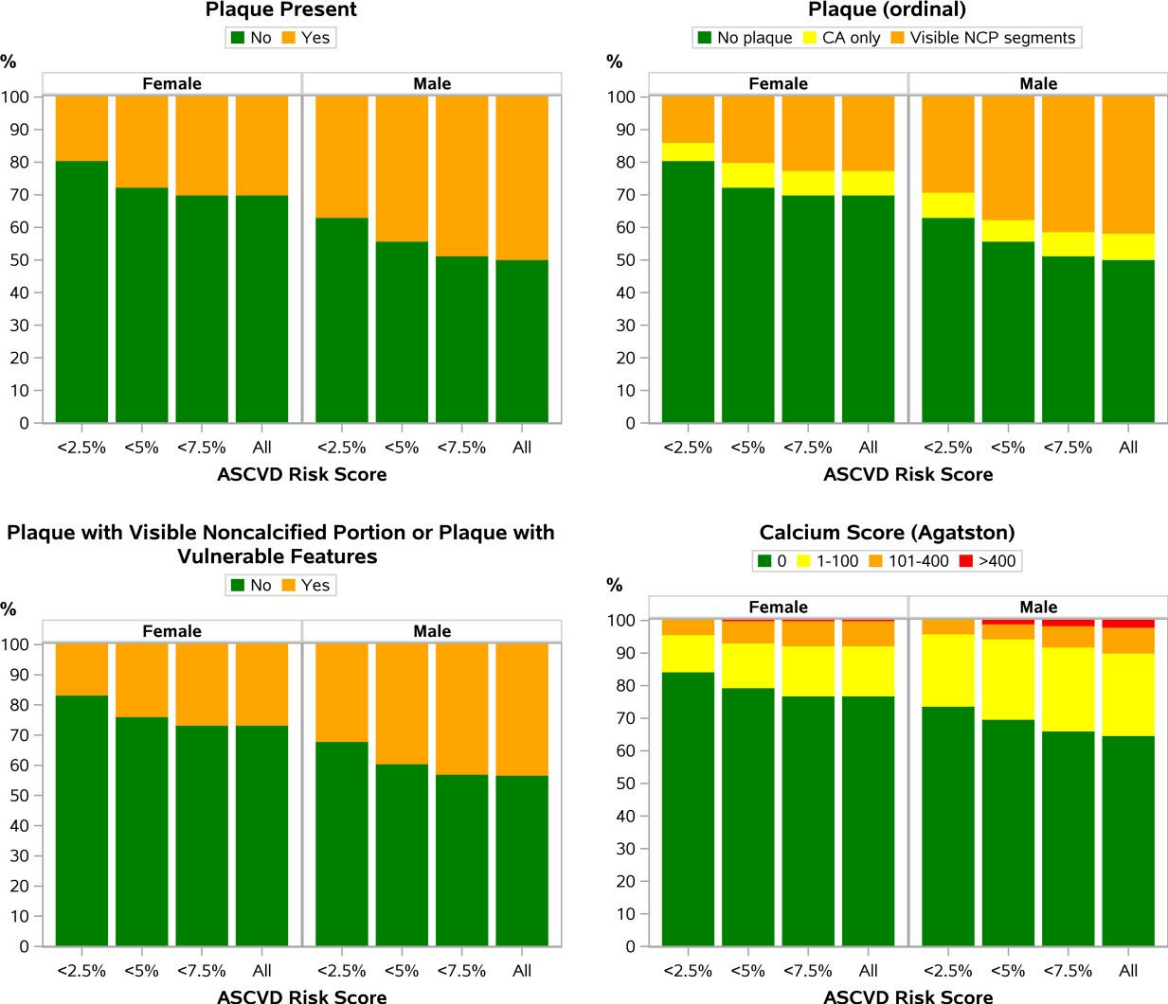
Prevalence of plaque outcomes by sex and ASCVD risk score. Among all participants, females (compared with males) had a lower prevalence of any plaque, NCP, VP, NC/V-P, and CAC >0. Females also had a lower prevalence of plaque, NCP, VP, NC/V-P, and CAC >0 when grouped by ASCVD risk score (eg, <2.5%, <5%, <7.5%— categories cumulative not mutually exclusive). Abbreviations: ASCVD, atherosclerotic cardiovascular disease; CA, calcified plaque; CAC, coronary artery calcium; NCP, noncalcified plaque; NC/V-P, noncalcified portion or vulnerable features; VP, vulnerable plaque feature.

**Figure 2.**
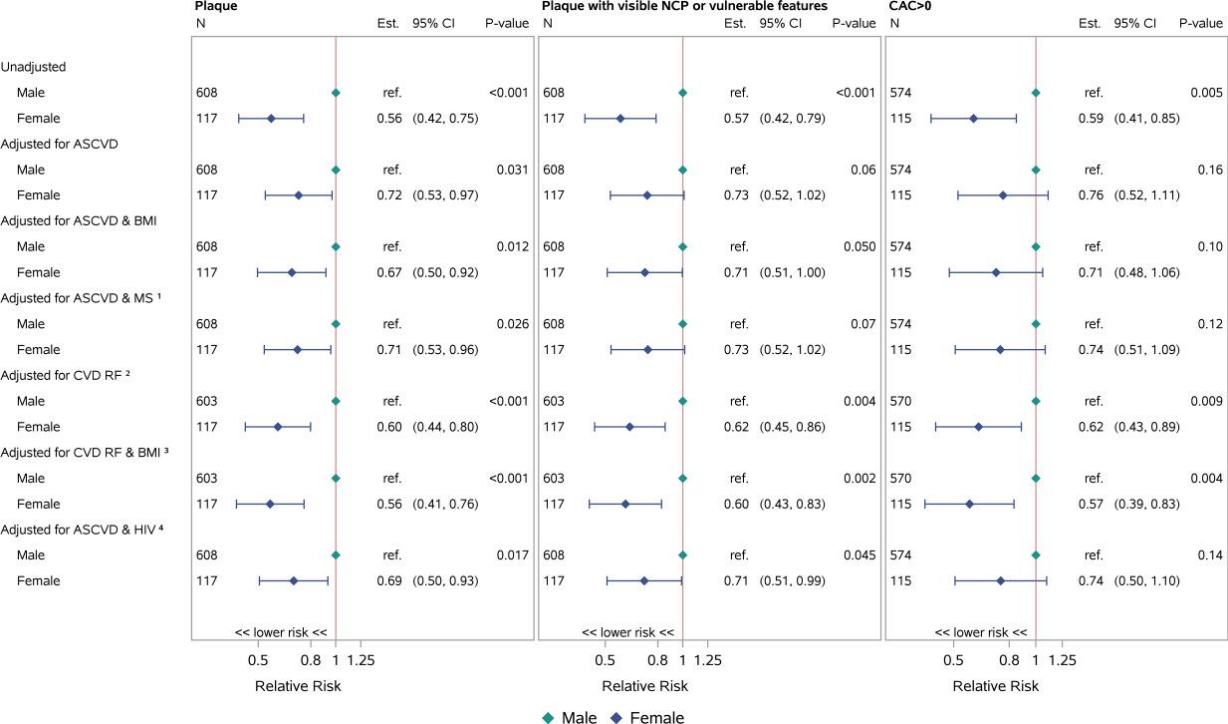
Risk of plaque outcomes (females compared with males). Restricted to participants with biomarker and flow data available. For females (compared with males), the prevalence of any plaque was lower in unadjusted analyses and in sequential analyses adjusting for ASCVD risk score, ASCVD risk score and BMI, ASCVD risk score and metabolic syndrome, individual CVD risk factors, individual CVD risk factors and BMI, or ASCVD risk score and HIV-specific risk factors. The prevalence of NC/V-P was lower in females in unadjusted analyses and in analyses adjusting for ASCVD risk score and BMI, individual CVD risk factors, individual CVD risk factors and BMI, or ASCVD risk score and HIV-specific risk factors. The prevalence of CAC >0 was lower among females in unadjusted analyses and in analyses adjusting either for individual CVD risk factors or for individual CVD risk factors and BMI. ^1^Metabolic syndrome was defined as presence of any 3 or more of elevated waist circumference, elevated triglycerides, reduced high HDL cholesterol, elevated blood pressure, and elevated fasting glucose. Sex- and population-specific thresholds were applied to classify waist circumference as elevated according to American Heart Association/National Heart Lung Blood Institute cut-points. ^2,3^CVD risk factors adjusted for include age, race, cigarette smoking, and HDL cholesterol. Models adjusting for hypertension and total cholesterol would not converge. ^4^HIV-specific risk factors included CD4 count, abacavir exposure, and protease inhibitor exposure. Abbreviations: ASCVD, atherosclerotic cardiovascular disease; BMI, body mass index; CAC, coronary artery calcium; CI, confidence interval; CVD, cardiovascular disease; Est., estimated; HIV, human immunodeficiency virus; MS, metabolic syndrome; NCP, noncalcified plaque; NC/V-P, noncalcified portion or vulnerable features; ref., reference; RF, risk factors.

Females (compared with males) had a lower prevalence of any plaque (31% vs 52%; *P* < .0001), NCP (23% vs 43%; *P* < .0001), VP (14% vs 25%; *P* = .008), NC/V-P (27% vs 45%; *P* = .0003), and CAC >0 (23% vs 37%; *P* = .003) (Table 2). Post hoc analysis revealed that the prevalence of plaque outcomes was lower among the subset of postmenopausal cis-gender females than among males (Supplementary Table 1). Females also had a lower prevalence of plaque, NCP, VP, NC/V-P, and CAC >0 when grouped by ASCVD risk score (eg, <2.5%, <5%, <7.5%—categories cumulative not mutually exclusive) (Figure 1). For females (compared with males), the prevalence of any plaque was lower in unadjusted analyses and in sequential analyses adjusting for ASCVD risk score, ASCVD risk score and BMI, ASCVD risk score and metabolic syndrome, individual CVD risk factors, individual CVD risk factors and BMI, or ASCVD risk score and HIV-specific risk factors. The prevalence of NC/V-P was lower in females in unadjusted analyses and in analyses adjusting for ASCVD risk score and BMI, individual CVD risk factors, individual CVD risk factors and BMI, or ASCVD risk score and HIV-specific risk factors. The prevalence of CAC >0 was lower among females in unadjusted analyses and in analyses adjusting either for individual CVD risk factors or for individual CVD risk factors and BMI (Figure 2). While the effects of increasing ASCVD risk levels on all plaque outcomes were consistently higher in females, these effects were estimated with low precision (wide CIs) and the differences were not statistically significant (*P-*interaction >.2 for all outcomes) (Table 3). In analyses restricted to those participants with any plaque (n = 368), no significant sex differences were appreciated in the prevalence of NC/V-P (Table 2).

**Table 2.** Prevalence of Plaque Outcomes, Overall and by Sex

	All Participants				Among Participants With Plaque			
	Total (N = 755)	Female (n = 124)	Male (n = 631)	*P*	Total (N = 368)	Female (n = 38)	Male (n = 330)	*P*
Plaque present								
Yes	368 (49%)	38 (31%)	330 (52%)	**<**.**0001**	368 (100%)	38 (100%)	330 (100%)	
Plaque with visible noncalcified portion								
Yes	302 (40%)	29 (23%)	273 (43%)	**<**.**0001**	302 (82%)	29 (76%)	273 (83%)	.33
Number of visible noncalcified segments								
ȃ0	453 (60%)	95 (77%)	358 (57%)	.**0002**	66 (18%)	9 (24%)	57 (17%)	.52
ȃ1–2	244 (32%)	23 (19%)	221 (35%)	**.0002**	244 (66%)	23 (61%)	221 (67%)	.52
ȃ≥3	58 (8%)	6 (5%)	52 (8%)	**.0002**	58 (16%)	6 (16%)	52 (16%)	.52
Plaque with vulnerable features								
Yes	172 (23%)	17 (14%)	155 (25%)	.**008**	172 (47%)	17 (45%)	155 (47%)	.79
Low attenuation plaque								
Yes	45 (6%)	2 (2%)	43 (7%)		45 (12%)	2 (5%)	43 (13%)	
Napkin ring sign								
Yes	23 (3%)	1 (1%)	22 (3%)		23 (6%)	1 (3%)	22 (7%)	
Positive remodeling								
Yes	166 (22%)	15 (12%)	151 (24%)		166 (45%)	15 (39%)	151 (46%)	
Plaque with visible noncalcified portion or plaque with vulnerable features								
Yes	318 (42%)	34 (27%)	284 (45%)	.**0003**	318 (86%)	34 (89%)	284 (86%)	.56
Calcium score (Agatston)								
>0	251 (35%)	28 (23%)	223 (37%)	.**003**	251 (72%)	28 (74%)	223 (72%)	.82
ȃNo. missing	37	3	34		20	…	20	
Among those with CAC >0								
1–100	177 (71%)	18 (64%)	159 (71%)	.65	177 (71%)	18 (64%)	159 (71%)	.65
101–400	61 (24%)	9 (32%)	52 (23%)	.65	61 (24%)	9 (32%)	52 (23%)	.65
>400	13 (5%)	1 (4%)	12 (5%)	.65	13 (5%)	1 (4%)	12 (5%)	.65

Prevalence is calculated out of those with the outcome available. Bold values represent a *P* value ≤0.05. Abbreviation: CAC, coronary artery calcium.

**Table 3.** Sex-Specific Relative Risk of Plaque Outcomes by ASCVD Risk Score

	Relative Risk (95% CI)	
ASCVD Risk Score	Females	Males
Plaque		
0–<2.5	ref.	ref.
2.5–<5	2.41 (1.28, 4.56)	1.30 (.96, 1.74)
5+	2.39 (1.17, 4.88)	1.62 (1.23, 2.14)
Plaque with visible NCP or vulnerable features		
0–<2.5	ref.	ref.
2.5–<5	2.41 (1.18, 4.93)	1.31 (.95, 1.81)
5+	2.83 (1.34, 5.98)	1.53 (1.13, 2.08)
CAC >0		
0–<2.5	ref.	ref.
2.5–<5	2.22 (1.01, 4.87)	1.26 (.84, 1.88)
5+	2.30 (.96, 5.48)	1.71 (1.18, 2.48)

Abbreviations: ASCVD, atherosclerotic cardiovascular disease; CAC, coronary artery calcium; CI, confidence interval; NCP, noncalcified plaque; ref., reference.

### Immune/Inflammatory Biomarker Outcomes: Overall and Sex-Stratified

Immune/inflammatory biomarker outcomes for the 725 participants with available paired data are shown in Table 4. Levels of biomarkers in the combined group of PWH are as previously reported [7]. The following sex differences in values of immune/inflammatory biomarkers were notable: on average, females (compared with males) had higher levels of IL-6, hs-CRP, and D-dimer and lower levels of Lp-PLA2 (all *P* < .001). These differences persisted after adjustment either for ASCVD risk score or for ASCVD risk score and BMI (Supplementary Figure 2). Small sex-based differences in the distributions of MCP-1 and sCD14 were noted; however, these were not apparent after adjustment for ASCVD risk score (Supplementary Figure 2).

**Table 4.** Levels of Immune/Inflammatory Biomarkers, Overall and by Sex

	Total (N = 725)	Females (n = 117)	Males (n = 608)	Group Difference
sCD14 (ng/mL)				
Median (Q1, Q3)	1817 (1528, 2176)	1883 (1592, 2248)	1772 (1494, 2163)	.021
P10, P90	1291, 2552	1400, 2712	1283, 2537	
No. missing	2	0	2	
sCD163 (ng/mL)				
Median (Q1, Q3)	840 (625, 1087)	893 (723, 1120)	829 (615, 1074)	.07
P10, P90	481, 1434	544, 1454	478, 1434	
No. missing	2	0	2	
MCP-1 (pg/mL)				
Median (Q1, Q3)	185 (146, 242)	171 (139, 231)	186 (149, 243)	.039
P10, P90	118, 310	111, 285	120, 312	
No. missing	3	0	3	
IL-6 (pg/mL)				
Median (Q1, Q3)	1.58 (0.99, 2.75)	2.07 (1.24, 3.29)	1.50 (0.95, 2.61)	**<**.**001**
P10, P90	0.75, 5.4	0.89, 5.8	0.74, 5.3	
No. missing	2	0	2	
hs-CRP (mg/L) (censored values imputed)				
Median (Q1, Q3)	1.75 (0.80, 3.60)	3.00 (1.50, 5.5)	1.60 (0.80, 3.20)	**<**.**001**
P10, P90	0.40, 8.5	0.50, 15.5	0.40, 7.3	
No. missing	3	0	3	
<1.0 mg/L	213 (30%)	20 (17%)	193 (32%)	**<**.**001**
1.0–3.0 mg/L	296 (41%)	41 (35%)	255 (42%)	
3.1–10.0 mg/L	153 (21%)	40 (34%)	113 (19%)	
>10.0 mg/L	60 (8%)	16 (14%)	44 (7%)	
Censoring^a^				
<0.3 mg/L	22 (3%)	3 (3%)	19 (3%)	
>10 mg/L	60 (8%)	16 (14%)	44 (7%)	
D-dimer (ng/mL)				
Median (Q1, Q3)	234 (143, 393)	340 (209, 558)	217 (134, 348)	**<**.**001**
P10, P90	97.0, 704	145, 926	93.0, 643	
No. missing	1	0	1	
Lp-PLA2 (ng/mL)				
Median (Q1, Q3)	130 (92.5, 168)	102 (65.5, 136)	135 (99.0, 174)	**<**.**001**
P10, P90	62.6, 207	49.6, 170	69.1, 214	
No. missing	2	0	2	
oxLDL (U/L)				
Median (Q1, Q3)	53.1 (42.1, 69.5)	52.6 (41.4, 67.7)	53.4 (42.2, 69.8)	.30
P10, P90	34.3, 88.9	33.5, 84.3	34.4, 89.4	
No. missing	2	0	2	

Abbreviations: hs-CRP, high-sensitivity C-reactive protein; IL-6, interleukin 6; Lp-PLA2, lipoprotein-associated phospholipase A2; MCP-1, monocyte chemoattractant protein-1; oxLDL, oxidized LDL; P, percentile; Q, quartile; sCD14, soluble CD14; sCD163, soluble CD163.

Censored values are imputed below the assay limit based on a uniform distribution.

### Immune–Plaque Relationships

**Figure 3.**
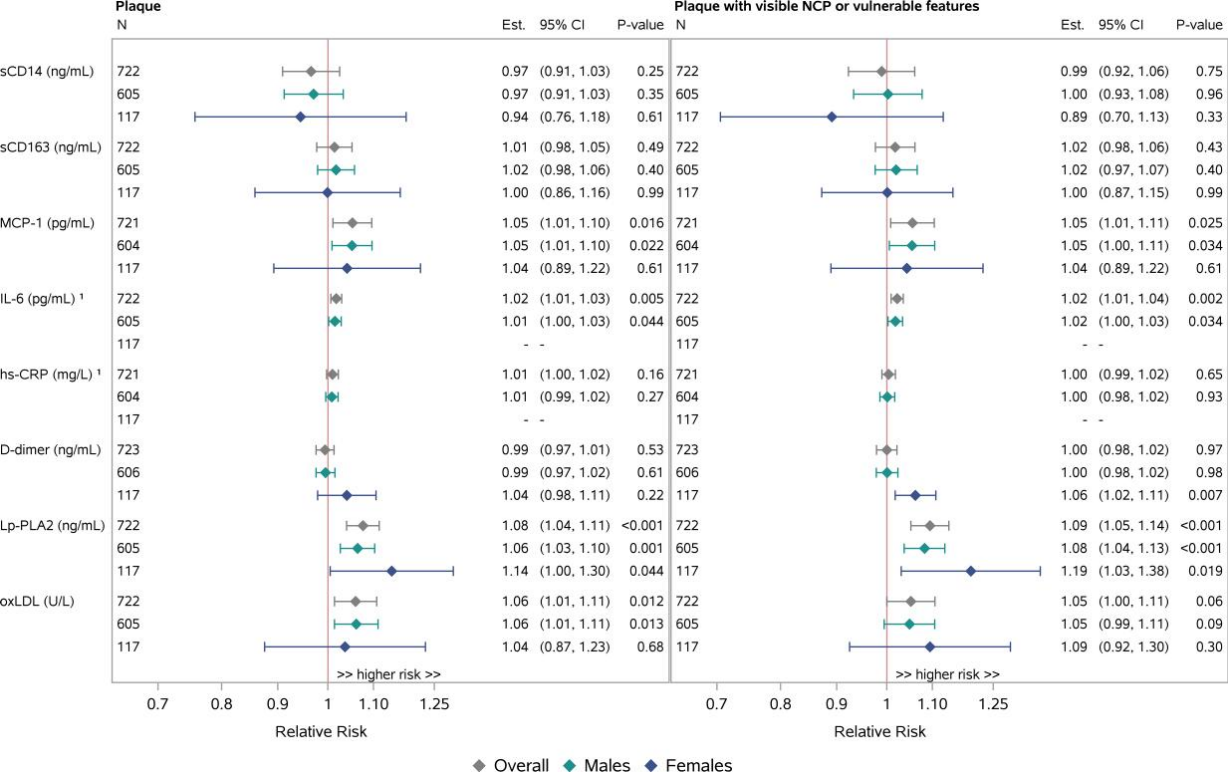
Immune–plaque relationships, adjusted log binomial regression in single biomarkers, overall and by sex. Adjusted for ASCVD risk score. Effect sizes are estimated per 25% increase in the biomarker. Notably, among females, but not among males, higher levels of D-dimer were associated with higher prevalence of NC/V-P. ^1^The validity of the model fit for IL-6 and hs-CRP is questionable for females. Abbreviations: ASCVD, atherosclerotic cardiovascular disease; CI, confidence interval; Est., estimated; hs-CRP, high-sensitivity C-reactive protein; IL-6, interleukin 6; Lp-PLA2, lipoprotein-associated phospholipase A2; MCP-1, monocyte chemoattractant protein-1; NCP, noncalcified plaque; NC/V-P, noncalcified portion or vulnerable features; oxLDL, oxidized LDL; sCD14, soluble CD14; sCD163, soluble CD163.

Relationships between immune/inflammatory biomarkers and coronary plaque outcomes (any plaque and NC/V-P) were assessed in the overall group and in groups stratified by sex (Figure 3). All analyses adjusted for ASCVD risk score.

In the overall group, higher levels of MCP-1, Lp-PLA2, and oxLDL were associated with a higher prevalence of plaque and NC/V-P. While a statistically significant relationship between higher levels of IL-6 and higher plaque or NC/V-P prevalence was noted, the magnitude of the effect size was small (Figure 3). Similar findings of higher levels of MCP-1, Lp-PLA2, and oxLDL associated with a higher prevalence of plaque and NC/V-P were apparent in sex-stratified analyses, although CIs were wide in the context of a smaller sample size for females (Figure 3). Apparent differences by sex included a larger effect of Lp-PLA2 on plaque outcomes among females, but formal tests for interaction by sex were not statistically significant (*P* > .20). Notably, among females but not among males, higher levels of D-dimer were associated with a higher prevalence of NC/V-P (1.06 [1.02, 1.11], *P* = .007 for females; 1.00 [.98, 1.02], *P* = .98 for males; *P*-interaction = .055] (Figure 3).

## DISCUSSION

**Figure 4.**
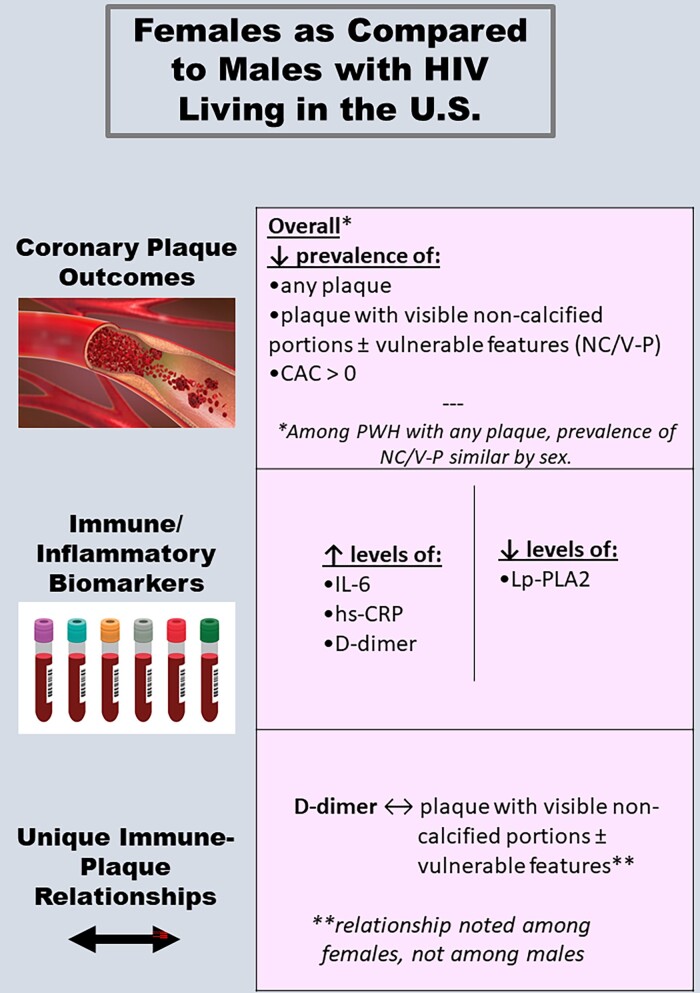
Central illustration highlighting sex differences in subclinical atherosclerosis and systemic immune activation/inflammation among with people with HIV in the U.S. Abbreviations: CAC, coronary artery calcium; HIV, human immunodeficiency virus; hs-CRP, high-sensitivity C-reactive protein; IL-6, interleukin 6; Lp-PLA2, lipoprotein-associated phospholipase A2; NC/V-P, noncalcified portion or vulnerable features.

Our analysis of coronary CTA and immune phenotypic data from a primary prevention cohort of ART-treated US PWH yielded key findings on sex differences in plaque, systemic immune/inflammatory biomarkers, and immune–plaque relationships. With respect to subclinical coronary atherosclerotic plaque, females had a lower prevalence of any plaque and NC/V-P across ASCVD strata. A lower prevalence of plaque and NC/V-P among females persisted after adjustment for traditional CVD risk factors. With respect to systemic immune/inflammatory biomarkers, females exhibited higher levels of IL-6, hs-CRP, and D-dimer and lower levels of Lp-PLA2, even after adjustment for ASCVD risk score. Finally, with respect to immune–plaque relationships, there were no differences by sex in the associations between higher levels of select immune/inflammatory biomarkers (MCP-1, Lp-PLA2, and oxLDL) and higher prevalence of plaque and/or NC/V-P. However, higher levels of the inflammatory/coagulation marker D-dimer were found to relate to a higher prevalence of NC/V-P exclusively among females (Figure 4).

Our findings of a lower prevalence of subclinical coronary plaque outcomes among US females with HIV dovetail with findings from studies engaging CVD-prevention cohorts of people without HIV [14, 15]. Moreover, our findings—derived from a large, contemporary cohort of ART-treated PWH from across the United States—build on previous findings from a small physiology study of PWH residing in the US Northeast [3]. However, the fact that our findings on a lower prevalence of plaque outcomes among females with HIV hold even after adjustment for CVD risk highlights an important question: If females both with and without HIV (vs males with and without HIV, respectively) are relatively protected from developing subclinical atherosclerotic plaque, why is the risk of HIV-associated MI among females double that among males in the United States [1]? Future studies could usefully interrogate 3 possibilities: first, that subclinical atherosclerotic plaque has uniquely high prognostic value for ensuing MI among females with HIV, potentially in relation to plaque erosion; second, that MI risk among females with HIV may be mediated through alternative pathways (eg, microvascular dysfunction), which are insufficiently characterized by CT-based evaluation of epicardial coronary arteries; or third, that females with HIV experience a disproportionate burden of events that predispose to myocardial oxygen supply/demand mismatch (eg, sepsis, overdose of illicit drugs, etc [2]).

In our study, effects of ASCVD risk levels on plaque outcomes tended to be higher in females. This finding suggests a need for attentive traditional CVD risk factor reduction in this population. Further, in analyses restricted to those PWH with any plaque, the prevalence of NC/V-P did not differ by sex. By contrast, general-population studies enrolling individuals with mild-to-moderate carotid artery stenosis have shown that females, compared with males, are less likely to have high-risk plaque features, even after adjustment for total plaque burden [16]. Given that vulnerable plaque morphology influences ensuing risk of clinical ASCVD events [17–19], our finding has potential clinical implications: among US females with HIV, prevalent coronary atherosclerotic plaque should not necessarily be inferred to reflect a more benign morphologic phenotype than that which is expected in males with HIV.

Among our cohort, female sex was associated with higher levels of IL-6, hs-CRP, and D-dimer and lower levels of Lp-PLA2, after adjustment for ASCVD risk score. Large-scale studies among people without HIV in the United States have revealed analogous patterns of sexual dimorphism in the expression of hs-CRP [20], D-dimer [21], and Lp-PLA2 [22], while sexual dimorphism in IL-6 expression has been suggested only through small-scale physiology studies [23]. Meanwhile, among PWH, analogous patterns of sexual dimorphism in some of these biomarkers have been suggested through large-scale US studies (IL-6 and CRP) [4] and international studies (D-dimer) [24]. To our knowledge, no prior large-scale study of PWH living in the United States has demonstrated an association between female sex and lower circulating levels of Lp-PLA2, after adjustment for ASCVD risk score. Importantly, the biomarkers characterized by sex-based expression differences in our study have been associated with incident CVD, severity of CVD outcomes, other non-AIDS complications, and all-cause mortality among PWH [4, 25–29].

A key component of our analysis plan entailed exploration for potential sex differences in relationships between circulating immune/inflammatory biomarkers and coronary atherosclerotic plaque parameters. There were no significant sex-based differences in relationships between select immune/inflammatory biomarkers (MCP-1, LpPLA2, and oxLDL) and the prevalence of coronary plaque and/or NC/V-P. However, of interest, higher levels of D-dimer were associated with a higher prevalence of NC/V-P among females with HIV and not among males with HIV. A prior analysis drawing from the CNICS cohort of PWH suggested that sex may modify the association between select systemic immune/inflammatory biomarkers and various cardiovascular events (eg, MI, stroke, venous thromboembolism, death) [4]. Meanwhile, analyses from the international Strategies for Management of Antiretroviral Therapy study showed relationships between D-dimer and CVD events, cardiovascular mortality, and non-cardiovascular mortality among PWH, but interactions for sex were not systematically assessed [26–29]. Ultimately, REPRIEVE will enable (1) identification of which systemic immune/inflammatory biomarkers are associated with adjudicated MACE in a global cohort of ART-treated PWH and (2) characterization of sex-based differences in the strength of observed immune–MACE relationships. These findings will facilitate interrogation of sex-based differences in statin-induced immunomodulation and statin-mediated ASCVD risk reduction among PWH.

Limitations of our study include the cross-sectional nature of the data, the regional specificity, and the reliance of plaque measures as surrogate risk markers for future ASCVD events. Additionally, our cohort of PWH included a greater percentage of males, limiting power to detect significant immune–plaque relationships in females. However, ours is one of the largest US cohorts of ART-treated males and females with HIV (recruited contemporaneously and under the same entry criteria) to have undergone both coronary CTA and immune phenotyping. Additional studies will be needed to explore the manner in which social determinants of health relevant to CVD risk [30] influence observed sex differences in subclinical atherosclerosis and immune activation/inflammation among US PWH. Such work will help ensure that recommended HIV-specific CVD-prevention strategies [31] are appropriately responsive to patients’ lived experience.

Overall, our study revealed that, among PWH living in the United States, females (compared with males) had a lower prevalence of subclinical coronary plaque and NC/V-P, as well as differences in key systemic immune/inflammatory biomarkers. Immune–plaque relationships differed by sex for D-dimer but not for other tested parameters. These points are informative given that sex differences in immune-mediated risks of CVD comorbidity may be driven either by differences in levels of select systemic immune/inflammatory biomarkers and/or by differences in the strength of relationships between systemic immune/inflammatory biomarkers and disease [32].

Our work advances understanding of potential sex-specific immune drivers of subclinical coronary pathology among ART-treated PWH and highlights the importance of performing sex-stratified analyses. Had sex-stratified modeling not been performed, the relationship between D-dimer and NC/V-P in this male-predominant cohort would have been obscured. Future investigations through REPRIEVE will assess sex specificity in immune predictors of MACE, statin-induced immunomodulation, and statin-mediated ASCVD risk reduction in this population. Taken together, the work will facilitate progress towards a more precise approach to immunomodulatory ASCVD risk reduction among PWH—an approach that accounts for sex as an important biologic variable.

## Supplementary Data

Supplementary materials are available at *Clinical Infectious Diseases* online. Consisting of data provided by the authors to benefit the reader, the posted materials are not copyedited and are the sole responsibility of the authors, so questions or comments should be addressed to the corresponding author.

## Supplementary Material

ciac767_Supplementary_Data

## Notes

***Acknowledgments.*** The study investigators thank the study participants, site staff, and study-associated personnel for their ongoing participation in the trial. Additionally, they thank the AIDS Clinical Trails Group (ACTG) for clinical site support; the ACTG Clinical Trials Specialists (Barbara Bastow, Laura Moran, Jhoanna Roa, and Nada Saleh) for regulatory support; the data management center, Frontier Science Foundation, for data support; and the Center for Biostatistics in AIDS Research for statistical support.

***Financial support.*** This work was supported through National Institutes of Health (NIH), National Institute of Heart, Lung, and Blood Institute (NHLBI) grants U01HL123336, to the REPRIEVE Clinical Coordinating Center, and U01HL123339, to the REPRIEVE Data Coordinating Center, as well as through funding from ViiV Healthcare, Kowa Pharmaceuticals, and Gilead Sciences. The National Institute of Allergy and Infectious Diseases (NIAID) supported this study through grants UM1AI068636, which supports the ACTG Leadership and Operations Center, and UM1AI106701, which supports the ACTG Laboratory. This work was also supported through NIAID grants R01AI123001 (to M. V. Z. and S. E. L.) and K24AI57882 (to M. V. Z.) and by NHLBI grant R01HL146267 (to M. V. Z.). M. V. Z. reports support as Co-Principal Investigator on an Investigator-Initiated Industry Grant from Gilead to the Principal Investigator’s institution (Massachusetts General Hospital).

***Potential conflicts of interest.*** M. V. Z. reports grant support through her institution from NIH/NIAID and Gilead Sciences, Inc, relevant to the conduct of the study, as well as grants from NIH/NIAID and NIH/NHLBI (RO1AI123001 Principal Investigator [PI]; R01 HL137562 PI; R01HL146267 PI; K24AI157882 PI; U01HL123336 Co-Investigator [Co-I]; U01HL123336-06S2 Co-I; R01HL151283 Co-I), support for attending meetings and/or travel for Conference on Retroviruses and Opportunistic Infections (CROI) and the International Workshop for HIV and Women from when abstract reviewer and/or speaker; and unpaid participation in Data Safety and Monitoring Boards (DSMBs) for NIH-funded studies, outside the submitted work. B. F. reports grant support through his institution from the NIH, Medimmune/AstraZeneca, and Medtrace, outside the submitted work. T. H. B. reports equity in Excision BioTherapeutics and service on their Scientific Advisory Board, outside the submitted work. S. E. L. reports travel reimbursement from the Association of Nurses in AIDS Care. C. D. M. reports personal fees from Gilead Sciences and ViiV Healthcare for participation in advisory board meetings, and grants to his institution from Gilead Sciences, outside the submitted work. C. J. F. reports grant support through his institution from Gilead Sciences, ViiV Healthcare, GSK, Janssen, AbbVie, Merck, Amgen, and Cytodyn, outside the submitted work, and personal fees from Theratechnologies and ViiV for consulting and participation on an Advisory Board unrelated to REPRIEVE with Theratechnologies and ViiV, and role as Chair on a DSMB for the Intrepid Study, outside the submitted work. E. T. O. reports grant support through his institution from NIH, Gilead, ViiV, and GSK; personal fees from Merck, Viiv Healthcare, and Theratechnologies; and positions as Chair of Comorbidity Transformational Science Group for the NIH-funded ACTG and Member of the Scientific Review Committee for NIH-funded HIV Vaccine Trials Network (HVTN), outside the submitted work. J. A. A. reports institutional research support for clinical trials from Atea, Emergent Biosolutions, Frontier Technologies, Gilead Sciences, GlaxoSmithKline, Janssen, Merck, Pfizer, Regeneron, and ViiV Healthcare and personal fees for advisory boards from GlaxoSmithKline/ViiV and Merck and participation on a DSMB for Kintor, all outside the submitted work. K. M. E. reports grant support from NIH/National Institute on Aging (NIA) and Gilead Sciences, and consulting fees from Gilead Sciences, ViiV Pharmaceuticals, and Janssen Therapeutics (all paid to the University of Colorado), and participation on a DSMB for an NIH study (paid to their institution), outside the submitted work. T. B. C. reports institutional research support from Gilead Sciences, ViiV, Regeneron, and Merck, as well as personal fees from ViiV for consulting, all outside the submitted work. J. S. C. reports consulting fees from Merck and Company (11/2021), paid to the author, and Resvirlogix, outside the submitted work. U. H. reports personal fees from Duke University, consulting fees from Recor Medical, and grant support from Kowa, AstraZeneca, Medimmune, and HeartFlow, outside the submitted work. M. T. L. reports grant support through his institution from Kowa Pharmaceuticals America, Inc, for the conduct of the study. He also reports grant support from MedImmune/AstraZeneca and personal fees from PQBypass, outside the submitted work. H. J. R. reports grant support from NIH/NIAID and NIH/NHLBI related to the conduct of the study, as well as grant support from NIH/NIAID, NIH/NHLBI, NIH/National Institute of Diabetes and Digestive and Kidney Diseases (NIDDK), and NIH/NIA, outside the submitted work. S. K. G. reports grant support through his institution from Kowa Pharmaceuticals America, Inc, Gilead Sciences, Inc, NIH, and ViiV for the conduct of the study, as well as grants from Theratechnologies and Navidea and personal fees from Theratechnologies Consulting and Viiv Consulting, and consulting fees from Navidea, all outside the submitted work. All other authors report no potential conflicts.
