# Supplementary material for: Sex Differences in Subclinical Atherosclerosis and Systemic Immune Activation/Inflammation Among People With Human Immunodeficiency Virus in the United States
Source: Clin Infect Dis. 2022 Sep 14;76(2):323–34. doi: 10.1093/cid/ciac767 (PMC9839188; doi:10.1093/cid/ciac767)
Supplement: ciac767_Supplementary_Data [file ciac767_supplementary_data.docx]

**Supplemental Appendix**

**Supplemental Figure 1. Algorithm Applied to Determine Post-Menopausal Status among Cis-gender Females**…………..…………………...2

**Supplemental Figure 2. Relationships between Sex and Levels of Immune/Inflammatory Biomarkers, Adjusted for ASCVD Risk** **Score or ASCVD Risk Score and BMI.** On average, females (compared with males) had higher levels of IL-6, hs-CRP and D-dimer and lower levels of Lp-PLA2. These differences persisted after adjustment. …………………………………………………………………………………………………………………………………………………………………………3

**Supplemental Table 1.** **Plaque Outcomes by Age Groups and by Menopausal Status among Cis-gender Females** ……………………………………………………………………………..…………………………………………………………………………………………4

**Supplemental Figure 1.** **Algorithm Applied to Determine Post-Menopausal Status among Cis-gender Females**


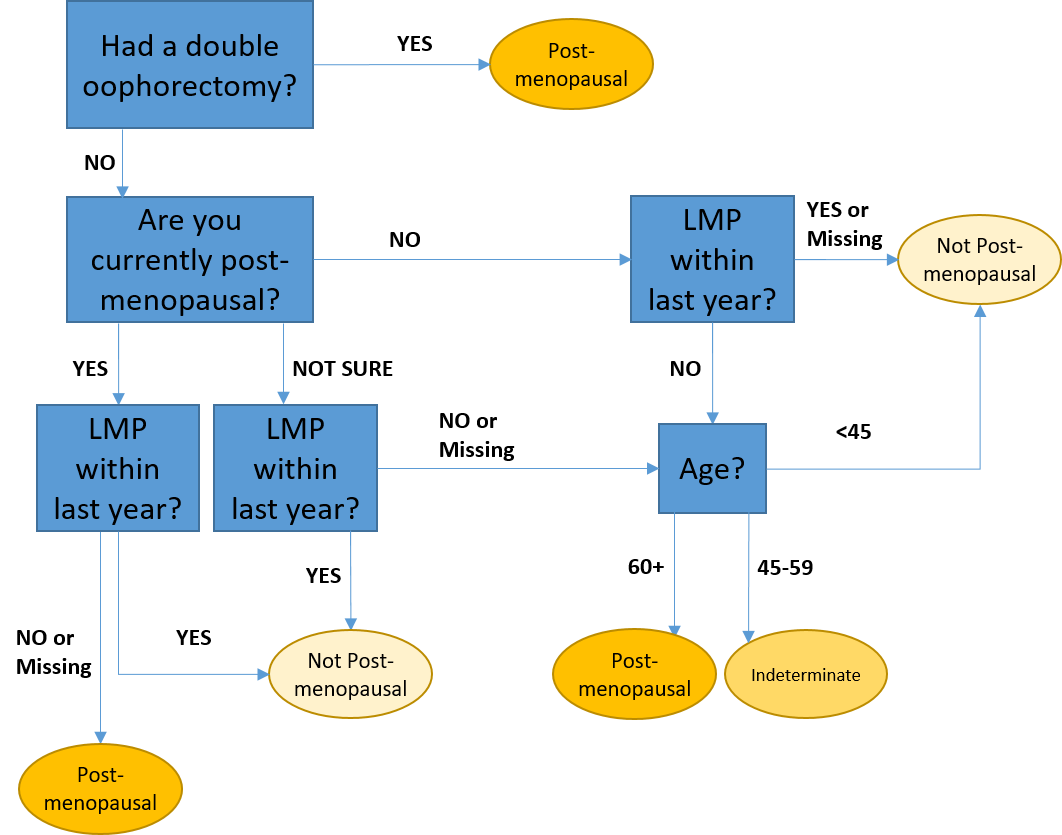


**Supplemental Figure 2. Relationships between Sex and Levels of Immune/Inflammatory Biomarkers**

| **a. Adjusted for ASCVD Risk Score** |
| --- |
| 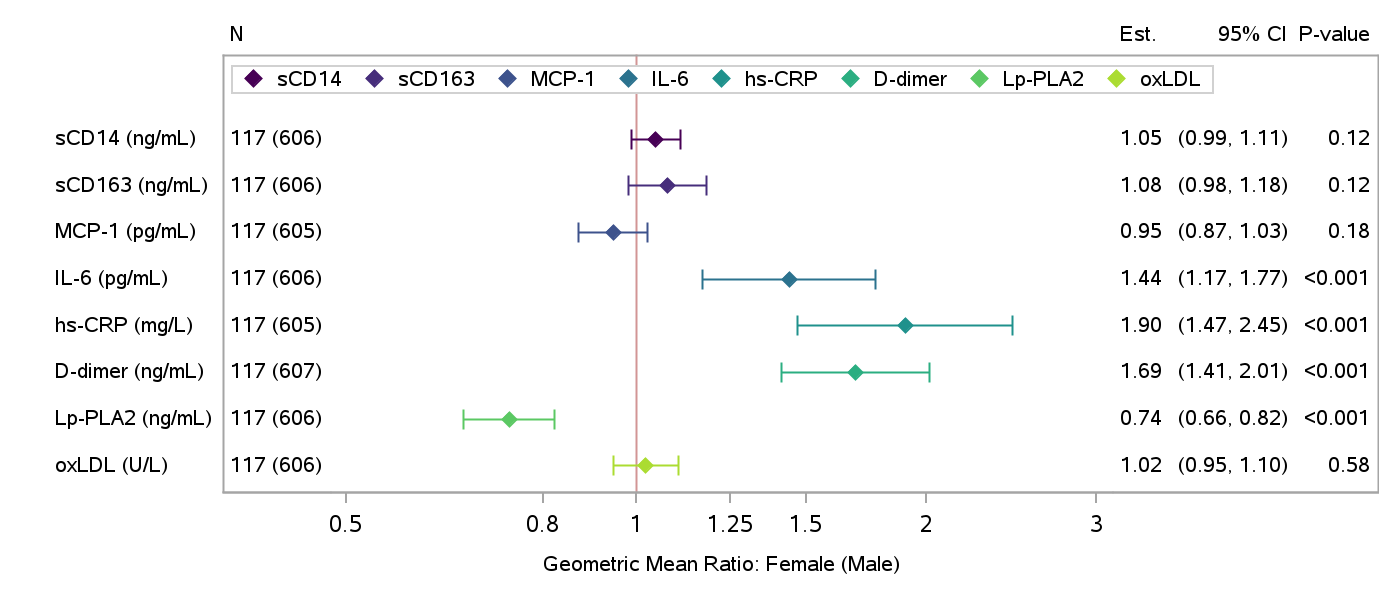 |

| **a. Adjusted for ASCVD Risk Score and BMI** |
| --- |
| 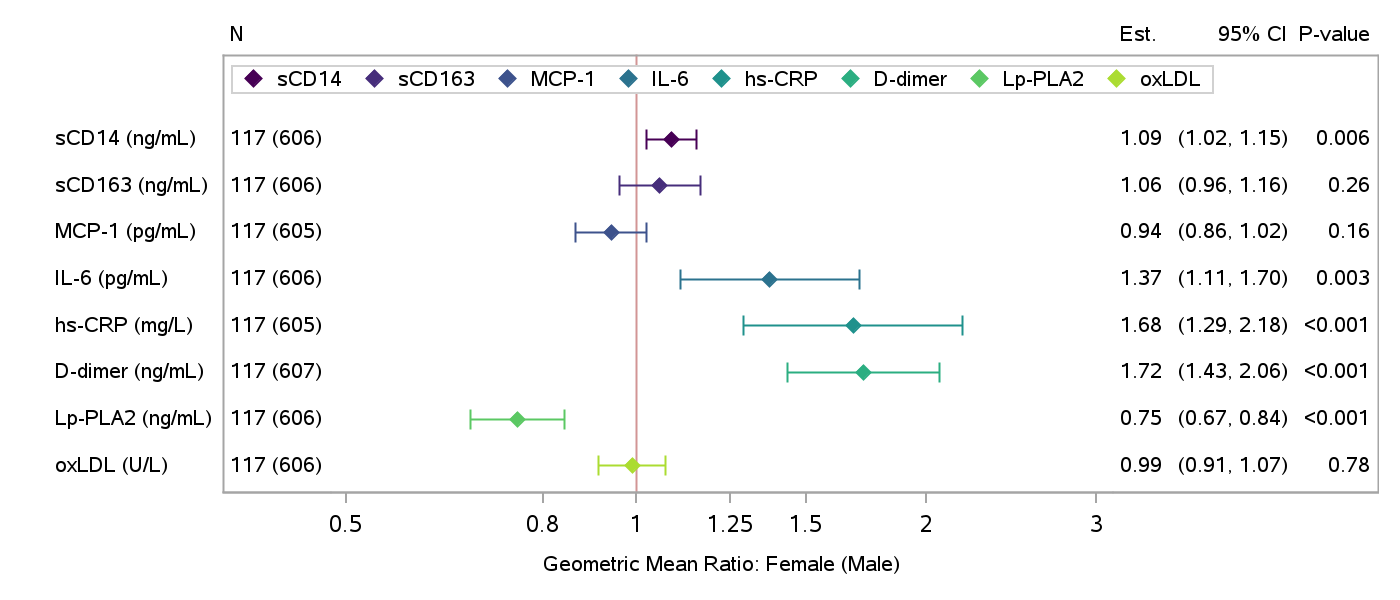 |

**Supplemental Table 1:** **Plaque Outcomes by Age Groups and by Menopausal Status among Cis-gender Females**

|  | | **Plaque** | | | **Plaque with visible NCP or vulnerable features** | | | **CAC>0** | | |
| --- | --- | --- | --- | --- | --- | --- | --- | --- | --- | --- |
| **Characteristic** |  | **Total (N=111)** | **No (N=77)** | **Yes (N=34)** | **Total (N=111)** | **No (N=81)** | **Yes (N=30)** | **Total (N=109)** | **No (N=83)** | **Yes (N=26)** |
| Age (years) | 40-49 | 49 | 39 (80%) | 10 (20%) | 49 | 42 (86%) | 7 (14%) | 48 | 40 (83%) | 8 (17%) |
|  | 50-59 | 52 | 32 (62%) | 20 (38%) | 52 | 33 (63%) | 19 (37%) | 51 | 37 (73%) | 14 (27%) |
|  | 60+ | 10 | 6 (60%) | 4 (40%) | 10 | 6 (60%) | 4 (40%) | 10 | 6 (60%) | 4 (40%) |
|  | | | | | | | | | | |
| Post-menopausal status | Post-menopausal | 59 | 34 (58%) | 25 (42%) | 59 | 38 (64%) | 21 (36%) | 59 | 38 (64%) | 21 (36%) |
|  | Pre-menopausal | 52 | 43 (83%) | 9 (17%) | 52 | 43 (83%) | 9 (17%) | 50 | 45 (90%) | 5 (10%) |
|  | | | | | | | | | | |

*Menopausal status is defined amongst cis-gender females only. Missing data (for menopausal status): Indeterminate (n=10).*
